# Cyclophilin E Functions as a Negative Regulator to Influenza Virus Replication by Impairing the Formation of the Viral Ribonucleoprotein Complex

**DOI:** 10.1371/journal.pone.0022625

**Published:** 2011-08-24

**Authors:** Zengfu Wang, Xiaoling Liu, Zhendong Zhao, Chongfeng Xu, Ke Zhang, Caiwei Chen, Lei Sun, George F. Gao, Xin Ye, Wenjun Liu

**Affiliations:** 1 Center for Molecular Virology, Chinese Academy of Sciences Key Laboratory of Pathogenic Microbiology and Immunology, Institute of Microbiology, Chinese Academy of Sciences, Beijing, China; 2 Graduate University of Chinese Academy of Sciences, Beijing, China; 3 China-Japan Joint Laboratory of Molecular Immunology and Molecular Microbiology, Institute of Microbiology, Chinese Academy of Sciences, Beijing, China; University of Cambridge, United Kingdom

## Abstract

**Background:**

The nucleoprotein (NP) of influenza A virus is a multifunctional protein that plays a critical role in the replication and transcription of the viral genome. Therefore, examining host factors that interact with NP may shed light on the mechanism of host restriction barriers and the tissue tropism of influenza A virus. Here, Cyclophilin E (CypE), a member of the peptidyl-propyl cis-trans isomerase (PPIase) family, was found to bind to NP and inhibit viral replication and transcription.

**Methodology/Principal Findings:**

In the present study, CypE was found to interact with NP but not with the other components of the viral ribonucleoprotein complex (vRNP): PB1, PB2, and PA. Mutagenesis data revealed that the CypE domain comprised of residues 137–186 is responsible for its binding to NP. Functional analysis results indicated that CypE is a negative regulator in the influenza virus life cycle. Furthermore, knock-down of CypE resulted in increased levels of three types of viral RNA, suggesting that CypE negatively affects viral replication and transcription. Moreover, up-regulation of CypE inhibited the activity of influenza viral polymerase. We determined that the molecular mechanism by which CypE negatively regulates influenza virus replication and transcription is by interfering with NP self-association and the NP-PB1 and NP-PB2 interactions.

**Conclusions/Significance:**

CypE is a host restriction factor that inhibits the functions of NP, as well as viral replication and transcription, by impairing the formation of the vRNP. The data presented here will help us to better understand the molecular mechanisms of host restriction barriers, host adaptation, and tissue tropism of influenza A virus.

## Introduction

Influenza virus belongs to the orthomyxoviruses and has a genome comprised of eight different negative strand RNAs varying in length from approximately 900 to 2,500 nucleotides [1]. Among the components of the influenza virion, the viral ribonucleoprotein complexes (vRNPs) are the viral core because they are independent units responsible for the replication and transcription of each virus segment. The native vRNPs are formed by single-stranded RNA, multiple monomers of the nucleoprotein (NP) and a single copy of the polymerase, a heterotrimer composed by the PB1, PB2, and PA subunits [2]. The NP of influenza A virus, encoded by segment 5, is a 498-amino acid polypeptide rich in arginine, glycine, and serine residues. In the virion, the NP forms the protein scaffold to package the helical genomic vRNPs [3], [4], [5]. The NP protein has multiple functions during the virus life cycle, and plays a critical role in influenza virus replication and transcription [6]. It interacts with viral macromolecules (including PB2, PB1, M1, and itself), displays high RNA binding activity, and binds to cellular factors to accomplish various functions, suggesting that NP is a key adapter molecule during the influenza virus infection cycle.

NP directly binds to two subunits of the viral RNA-dependent RNA polymerase, PB1 and PB2, but not with PA, both in virus-infected cells and recombinant systems in the absence of viral RNA [7], [8]. The cryo-electron microscopy structure of purified biologically active recombinant vRNPs shows that NP monomers form a nonameric ring, indicating that the homo-oligomerization of NP plays an important role in maintaining the vRNP structure for influenza virus replication and transcription [4], [9], [10]. Although the NP of influenza A virus displays high affinity for RNA but little or no sequence specificity, it cannot protect RNA from RNase digestion [4], [10], [11]. NP can bind any type of RNA longer than 15 nucleotides and displays no higher binding affinity for influenza virus-specific RNA sequences [12].

Several host factors interact with NP, such as importin-α, F-actin, exportin 1 (CRM1), and nuclear factor 90 (NF90) [13], [14], [15], [16]. The interaction between NP and importin α is thought to be a determinant of the host range of influenza A virus [14]. Moreover, the interaction between PB2 and NP with different importin-α isoforms governs the host adaptation or cell tropism of influenza A virus [17], [18]. NP further mediates the nuclear import of vRNPs through its interaction with importin α [15]. The interaction between NP and F-actin causes modulation of NP localization in the nucleus of infected cells [13], and NP nuclear export is mediated by a direct interaction between NP and CRM1 [19].

The above host factors are only associated with NP nuclear shuttling, while another host factor, NF90, is involved in regulating the replication and transcription of influenza A virus. NF90 negatively regulates viral replication and transcription during the early phase of infection by directly interacting with the NP of influenza A virus [16]. Recently, a cellular deubiquitinase, USP11, was found to inhibit the RNA replication of influenza A virus by specifically deubiquitinating NP [20].

Many of the cyclophilins, such as cyclophilin A (CypA) and cyclophilin B (CypB), interact with various viral proteins and are involved in viral life cycles. However, the mechanisms by which cyclophilins are involved in viral life cycles are often unclear. CypA interacts with the gag protein of human immunodeficiency virus (HIV), package in HIV virions and benefit for virus replication [21], [22]. In addition, CypA is as an essential cofactor for hepatitis C virus infection by mediating viral cyclosporine resistance [23]. CypA also interacts with the M1 protein of influenza A virus and inhibits virus replication during the early stage of viral infection [24], [25]. The interaction between CypB and NS5B is essential for hepatitis C virus replication [26].

In the present study, CypE, a nuclear and RNA-binding cyclophilin [27], was identified to interact with NP. Several lines of evidences indicate that CypE serves as a host restriction factor that targets the functions of NP. Moreover, we found that the molecular mechanism by which CypE negatively regulates influenza virus replication and transcription occurs by impairing the formation of the vRNP.

## Results

### CypE interacts with influenza A virus NP

**Figure 1:**
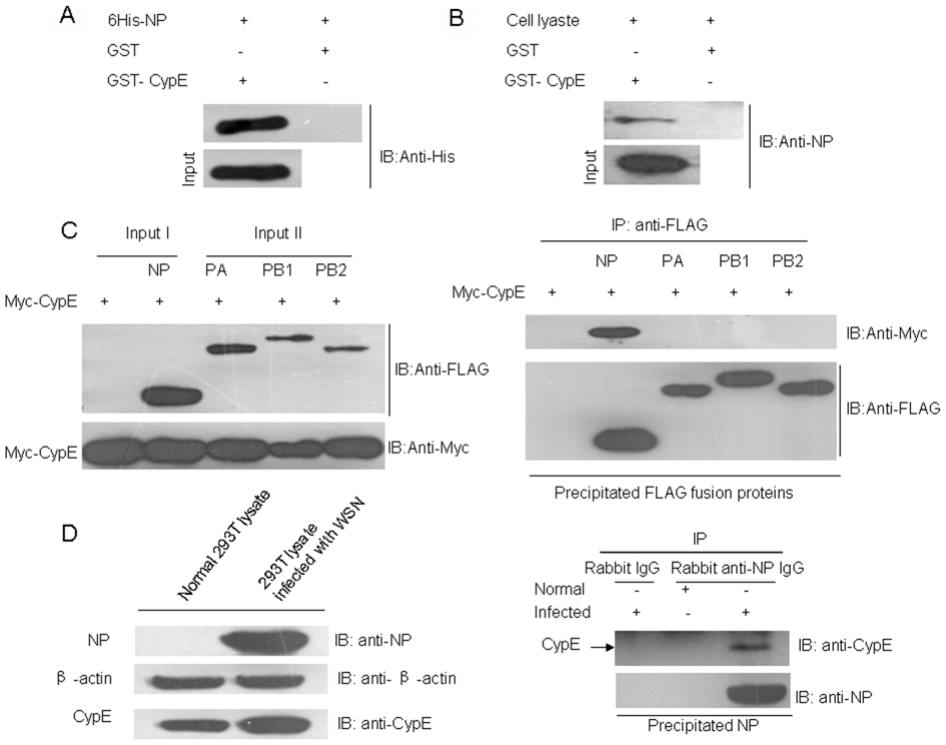
CypE interacts with the NP of influenza A virus. (A, B) His-NP fusion protein and lysates from MDCK cells infected with influenza A virus (A/WSN/33) were incubated with an equal amount of GST alone or GST-CypE bound to glutathione-Sepharose 4B beads. After extensive washing, the proteins bound to the beads were extracted and analyzed by western blotting with the indicated antibodies. (C) FLAG-tagged NP, PA, PB1, or PB2 plus Myc-tagged CypE were transfected into 293T cells. The cell lysates were immunoblotted with anti-Myc or anti-FLAG antibodies to confirm the expression of the proteins of interest in the 293T cells. The cell lysates were subjected to co-immunoprecipitation assays using anti-FLAG M2 affinity gel. The immunoprecipitated proteins were assayed with an anti-Myc polyclonal antibody. “Input I” and “Input II” show approximately 1/10 and 1/20 of the total protein, respectively. (D) The 293T cells were infected with A/WSN/33 (MOI = 1). At 12 h p.i., the cell lysates were subjected to co-immunoprecipitation assay using rabbit anti-NP antibody.

Because NP is located in the nucleus during viral replication and interacts with several nuclear proteins, we investigated whether the nuclear protein CypE is involved in influenza virus replication via a direct interaction with NP. To identify if NP interacts with CypE, GST pull-down assays were performed using GST-CypE and His-NP fusion proteins or cell lysate from MDCK cells infected with influenza A virus (A/WSN/33). As shown in **Figure 1A**, GST-CypE interacted with purified NP, as well as the NP from cells infected with influenza A virus (**Figure 1A and B**).

To examine whether CypE interacts with NP *in vivo*, 293T cells were transfected with Myc-tagged CypE and FLAG-tagged NP, PB1, PB2, or PA, and the cell lysates were subjected to immunoprecipitation analysis. The data demonstrate that Myc-tagged CypE only interacted with FLAG-tagged NP and not PA, PB1, or PB2 (**Figure 1C**). To further confirm the interaction between NP and CypE, we performed binding assays to determine if endogenous CypE interacts with NP during viral infection. As shown in **Figure 1D**, NP bound to endogenous CypE, indicating that CypE could affect virus replication through its interaction with NP.

**Figure 2:**
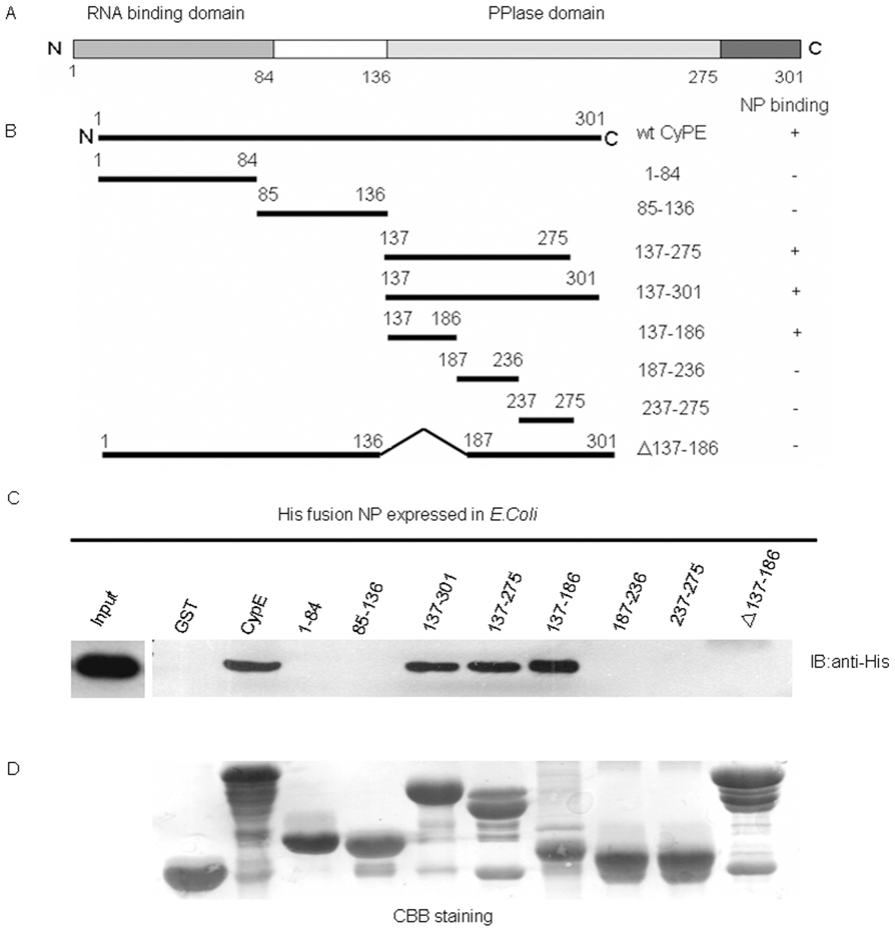
Mapping the domain of CypE responsible for the interaction with NP. (A) CypE has two functional domains: an RNA-binding domain at its N-terminus (aa 1–84) and a PPIase domain at its C-terminus (aa 136–275). (B) The binding regions on CypE for NP were confirmed by GST pull-down assays. “NP binding” summarizes the results of the GST pull-down assays. (C) GST pull-down assays were performed using CypE and its truncation mutants. “Input” shows 1/20 of the total protein included in each binding reaction. (D) Coomassie brilliant blue (CBB) staining patterns for the pull-down proteins are shown.

To determine which domain of CypE is responsible for binding to NP, we generated several truncated mutants of GST-CypE (**Figure 2A and B**). GST pull-down assays were then performed using GST-CypE and its truncated mutants. As shown in **Figure 2B and C**, among the CypE mutants, only the truncation of CypE between residues 137–186 in the PPIase domain was sufficient and required for its interaction with NP. These data suggest that residues 137–186 in the CypE PPIase domain comprise the critical region for NP binding.

**Figure 3:**
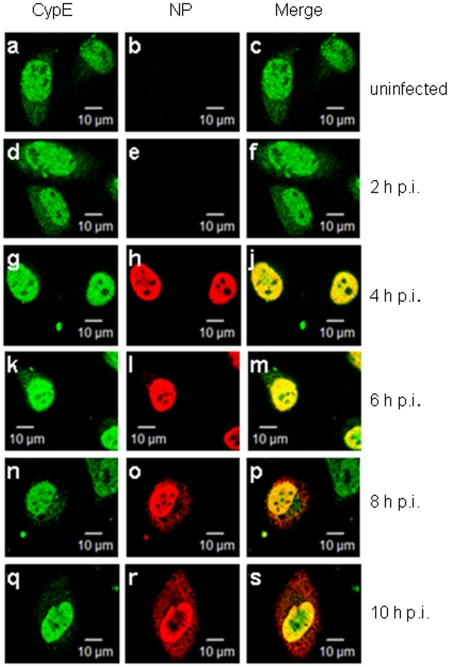
CypE co-localizes with NP during influenza virus infection. The localization of CypE and NP was determined by immunofluorescence assays using anti-CypE and anti-NP polyclonal antibodies from 2 to 10 h p.i. at 2 h intervals. Scale bar: 10 µm.

As is well known, NP localizes to the nucleus and/or cytoplasm during different infection stages. Accordingly, the intracellular co-localization of CypE and NP was examined during viral infection at 2 h intervals. HeLa cells were infected with A/WSN/33 and immunostained with anti-CypE and anti-NP antibodies. The immunofluorescence data revealed that both CypE and NP co-localize in the nucleus from 4 to 8 h post-infection (p.i.), while NP is predominantly located in the cytoplasm (and there is correspondingly less co-localization of CypE and NP in the nucleus) at 10 h p.i. (**Figure 3**). These data suggest that the interaction between CypE and NP occurs in the early phase of viral replication.

### CypE negatively regulated influenza A virus replication

**Figure 4:**
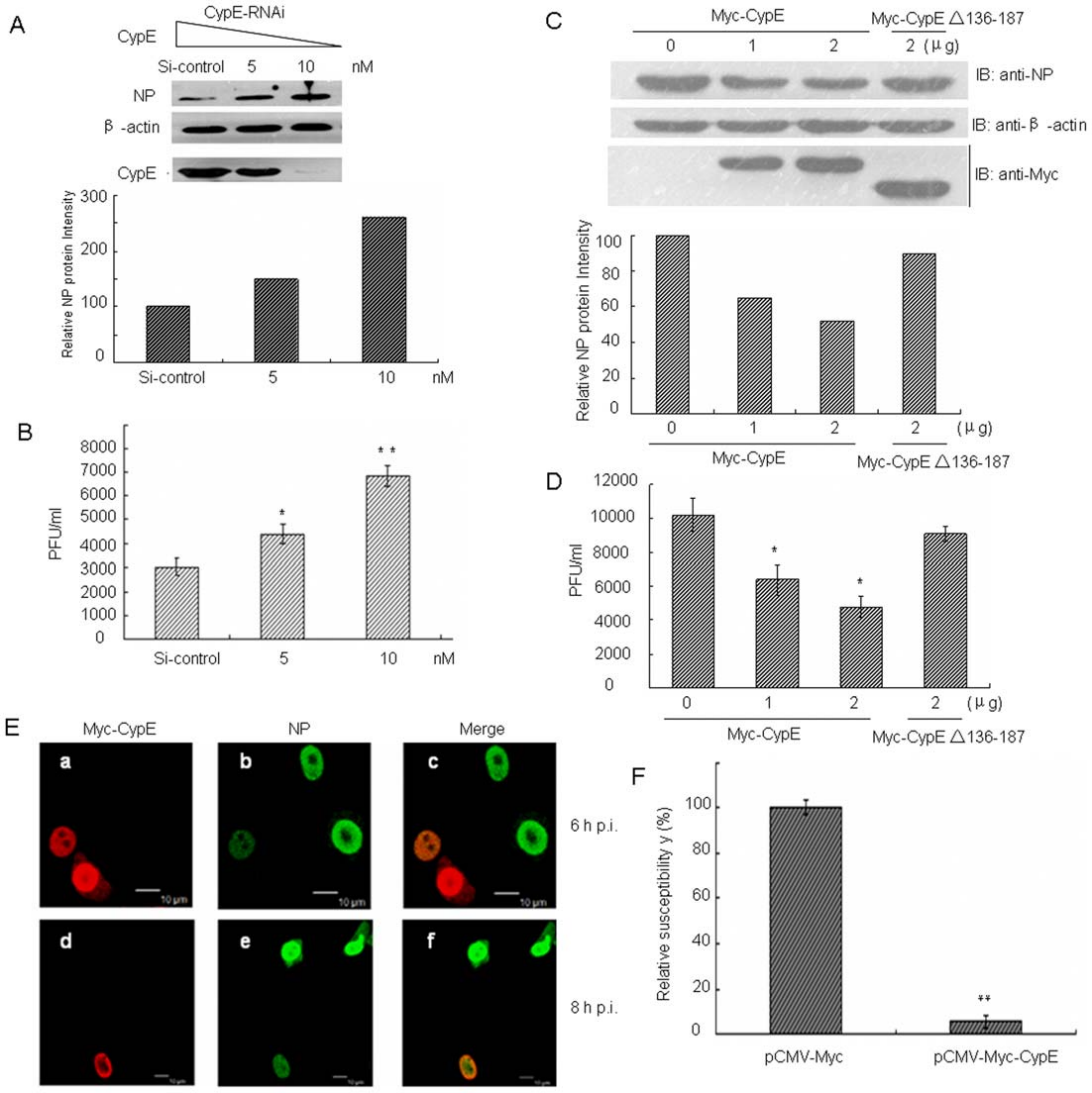
CypE inhibits influenza A virus replication. (A–D) As described in the Materials and Methods, 293T cells were transfected with si-CypE, Myc-CypE, or Myc-CypE Δ136–187 plasmid and then infected with influenza virus A/WSN/33 at an MOI of 0.1. The cell lysates were analyzed by western blotting with anti-CypE, anti-β-actin, and anti-NP antibodies (A, C). The media were collected, and the viral titers were measured (B, D). Error bars represent the standard deviation (SD). (E) Over-expression of CypE decreases the infectivity of influenza virus. The 293T cells were transfected with pCMV-Myc empty plasmid or pCMV-Myc-CypE. At 12 h p.t., the cells were infected with A/WSN/33 virus (MOI = 1). At 6 and 8 h p.i., the cells were fixed and then stained with anti-NP antibody and anti-Myc antibody, respectively. Scale bars: 10 µm. (F) The 293T cells were transfected with pCMV-Myc empty plasmid or pCMV-Myc-CypE, together with pEGFP-N1 (10∶1). At 12 h p.t., the cells were infected with A/WSN/33 virus (MOI = 1) for 4 h and then stained for NP. The percent of red color (TRITC stain) in EGFP-expressing cells was calculated, and this was the infectivity of influenza virus. The data represent the means of three independent experiments. Error bars represent the SD. *, p<0.05. **; and p<0.01.

To investigate the biological effect of CypE on influenza A virus replication, we analyzed virus replication in the 293T and A549 cell lines where CypE was knocked down by RNA interference. As shown in **Figure 4A** and **Figure S1**, the level of NP was significantly increased (∼2.5-fold) in the CypE knock-down cells compared to the control cells. The virus titer was also increased ∼2.5-fold in endogenous CypE-depleted cells (**Figure 4B** and **Figure S1**). These results indicate that knock-down of endogenous CypE favors the replication of influenza A virus. To further analyze the function of CypE on viral replication, 293T cells were transfected with Myc-CypE or Myc-CypE Δ137–186, followed by infection with A/WSN/33. Consistently, the NP level was greatly decreased in CypE over-expressing cells but not in CypE Δ137–186 over-expressing cells (**Figure 4C**), suggesting that the antiviral function of CypE is dependent on its binding to NP. In addition, the virus titer in CypE over-expressing cells was decreased approximately twofold compared to the control cells (**Figure 4D**). Taken together, it is clear that CypE exhibits an inhibitory effect on viral replication.

To examine the effect of CypE on the infectivity of influenza virus, 293T cells were transfected with pCMV-Myc-CypE for 12 h and then infected with A/WSN/33. The cells were subsequently immunostained with anti-NP and anti-Myc antibodies. As shown in **Figure 4E**, a portion of NP translocated into the cytoplasm from the nucleus in the control cells at 8 h p.i., though most of the NP was still located in the nucleus and co-localized with over-expressed CypE. However, the amount of NP in the CypE over-expressing cells was much less than that in the control cells (**Figure 4E a–f**). Furthermore, the relative susceptibility of 293T cells to viral infection was reduced to ∼10% in CypE over-expressing cells compared to control cells (**Figure 4F**). These data indicate only a 2- or 2.5-fold difference in virus growth in the presence or absence of CypE. In fact, the role of CypE in influenza virus replication may be significant if 100% of the transfected cells were CypE over-expressing or knocked-down, but the inhibitory efficiency of CypE was not comparable to that of anti-influenza drugs.

### CypE inhibits the transcription and replication of influenza virus

**Figure 5:**
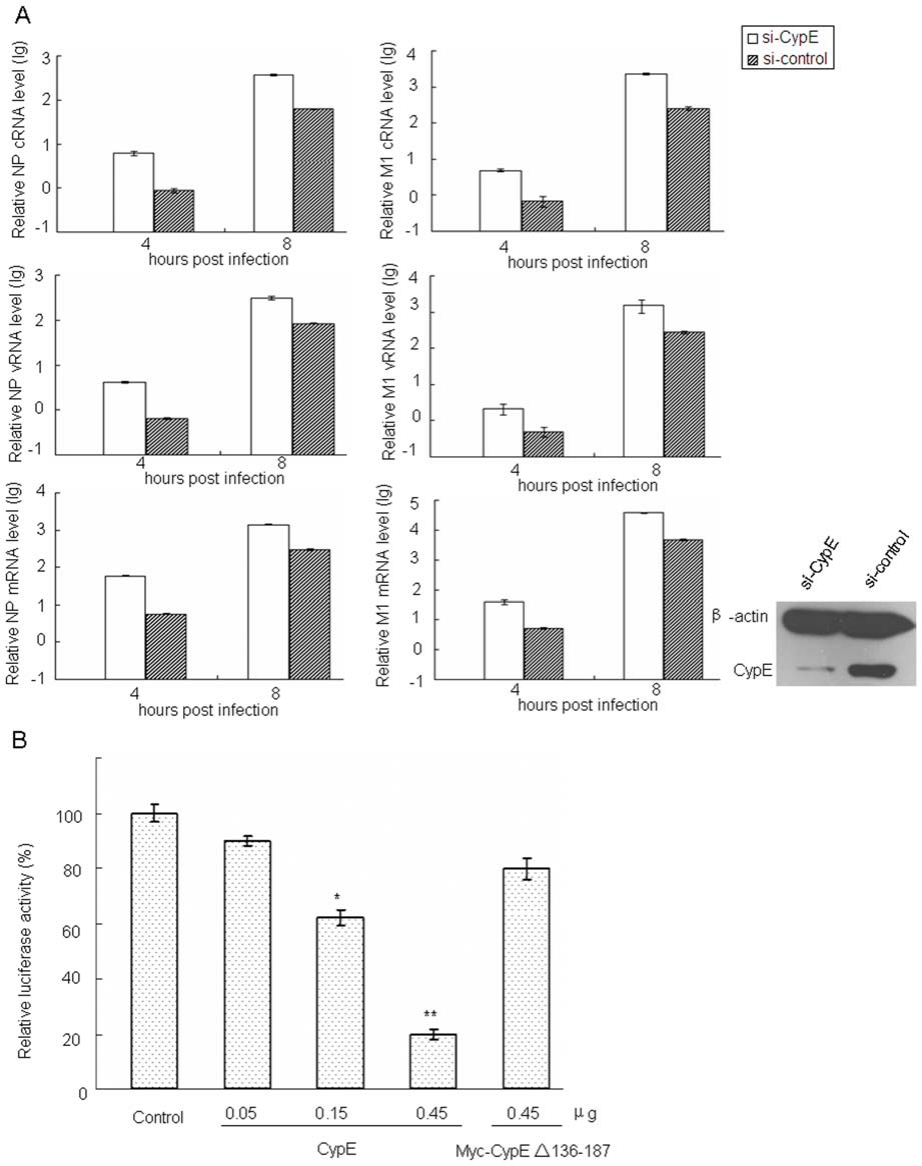
CypE inhibits the transcription and replication of influenza virus. (A) The A549 cells were treated with si-CypE or si-control for 48 h and then infected with influenza virus A/WSN/33 at an MOI of 1. Three types of RNA levels of the NP and M1 genes were analyzed with or without CypE-knock-down by quantitative real-time PCR after viral infection (4 and 8 h). Western blot to confirm the knock-down of CypE expression in A549 cells. Error bars represented the standard error of the mean (SEM). (B) All polymerase complex component plasmids and the luciferase gene, along with different doses of CypE and Myc-CypE Δ136–187 plasmids, were transfected into 293T cells, respectively. The luciferase activity in triplicate cultures was estimated and compared at 36 h p.t.. *, *p<0.05*; and **, *p<0.01*.

To further understand the effect of CypE on the replication and/or transcription of influenza A virus, three types of RNA levels (viral RNA (vRNA), complementary RNA (cRNA), and mRNA) of the NP and M1 genes were detected with or without CypE knock-down by quantitative real-time PCR 4 and 8 h after viral infection. The amount of the three types of RNA increased (∼5–12 fold) in the CypE knock-down cells compared to the silencing control cells (**Figure 5A**). In contrast, levels of the three types of RNA decreased (∼4–8 fold) in the CypE over-expressing cells compared to control cells (**Figure S2**). The present data indicate that CypE is a negative regulator both at the replication and transcription levels in the viral life cycle.

To examine if CypE affects the activity of influenza virus polymerase, 293T cells were transfected with Myc-CypE and the vRNP genes (NP, PB1, PB2 and PA) derived from A/WSN/33, along with a reporter plasmid containing non-coding sequence from the NS segment of the influenza A virus genome and the luciferase gene driven by the PolI promoter. We found that the vRNP activity was decreased in the CypE over-expressing cells compared to the control cells, indicating that the over-expression of CypE inhibits viral replication and transcription by impairing vRNP activity. Additionally, the inhibitory effect of CypE on vRNP activity occurred in a dose-dependent manner (**Figure 5B**). Though the CypE Δ137–186 mutant had a minor negative effect on virus polymerase activity, this difference was not significant compared to the control by T-test.

### CypE impairs the formation of vRNP

**Figure 6:**
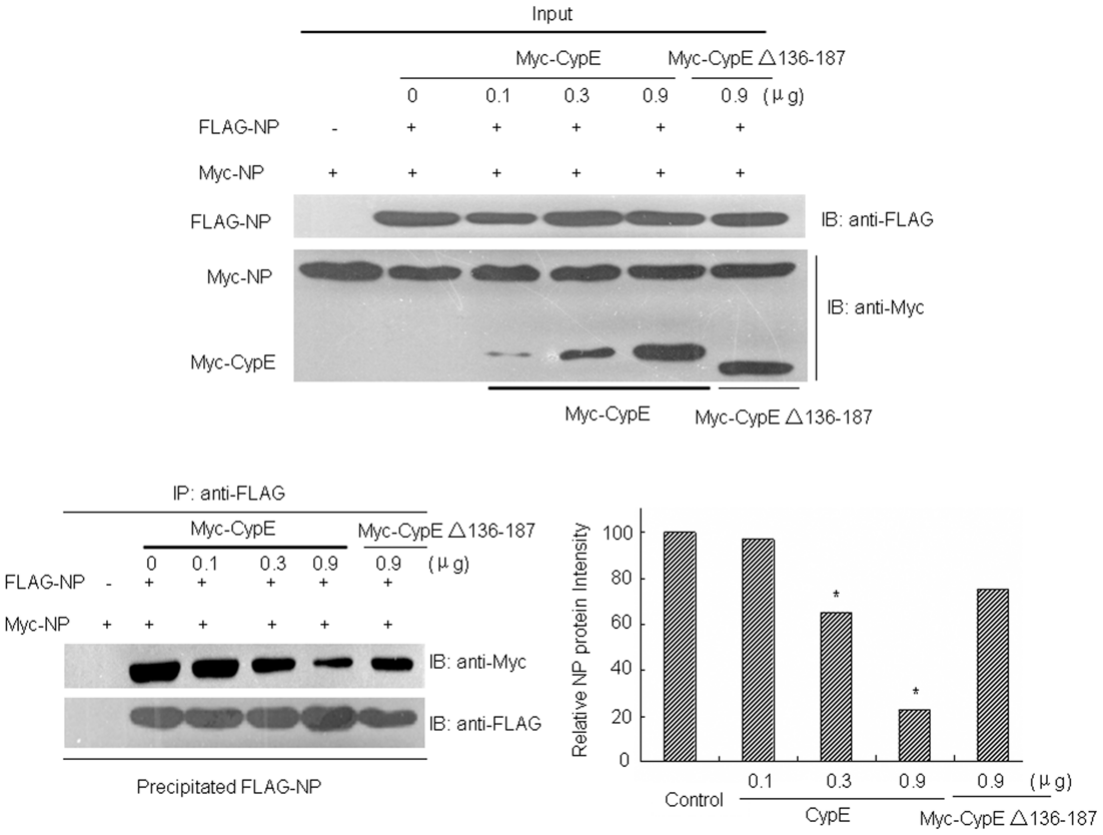
CypE inhibits NP self-association. pCDNA3-FLAG-NP and pCMV-Myc-NP, together with different doses of pCMV-Myc-CypE or Myc-CypE Δ136–187 plasmids, were transfected into 293T cells. At 48 h p.t, the cell lysates were immunoblotted with anti-Myc or anti-FLAG antibodies to confirm the expression of the proteins of interest in the 293T cells. Co-immunoprecipitation assays were then performed using anti-FLAG M2 affinity gel. The immunoprecipitated proteins were assayed with an anti-Myc antibody. “Input” shows ∼1/10 of the total protein. *, p<0.05.

It has been reported that the oligomerization of NP is critical for the replication and transcription of influenza A virus [28], [29], [30]. To analyze whether CypE affects the oligomerization of NP, 293T cells were transfected with both Myc- and FLAG-tagged NP expression plasmids, together with Myc-tagged CypE or CypE Δ137–186. Cell lysates were then immunoprecipitated with FLAG antibody and immunoblotted with Myc antibody to detect precipitated Myc-NP (**Figure 6**). We found that CypE inhibited NP self-association in a dose-dependent manner, while the CypE Δ137–186 mutant had very little influence on the NP-NP interaction (**Figure 6**). Thus, CypE blocks the formation of vRNP by inhibiting NP self-association.

**Figure 7:**
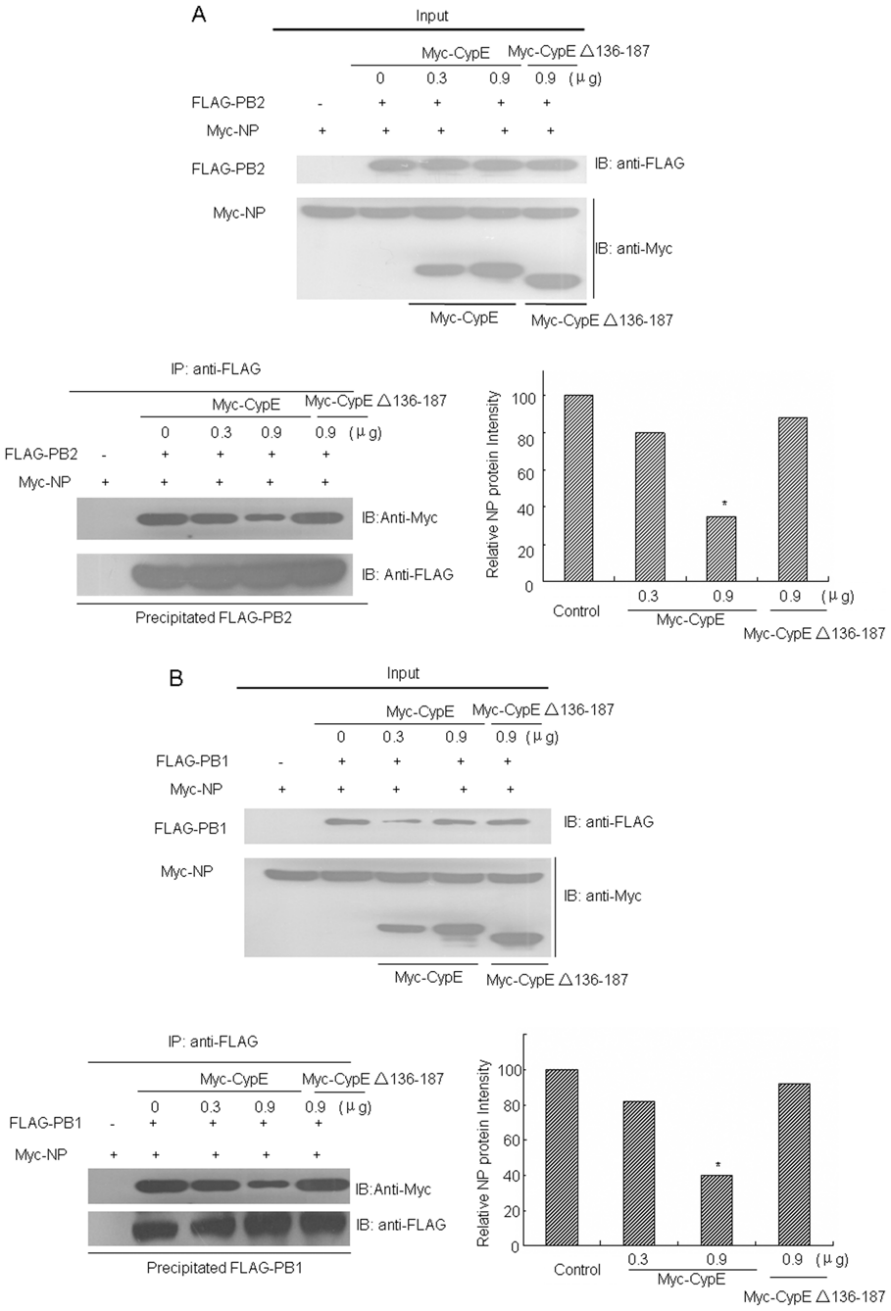
CypE impairs the interaction of NP with PB1 and PB2. The pCDNA3-FLAG-PB1 or pCDNA3-FLAG-PB2 and pCMV-Myc-NP plasmids, along with pCMV-Myc-CypE (0, 0.3, and 0.9 µg) or Myc-CypE Δ136–187 (0.9 µg), were transfected into 293T cells, respectively. At 48 h p.t, the cells were lysed, immunoblotted with anti-Myc or anti-FLAG antibodies, and subjected to co-immunoprecipitation assays. The immunoprecipitated NP was detected with anti-Myc antibody. “Input” shows ∼1/20 of the total protein. *, p<0.05.

Because the interaction between NP and polymerase proteins is critical for regulating the switch of vRNA synthesis from transcription to replication [8], it is important to understand if CypE affects the NP-PB1 and NP-PB2 interactions. To examine this, 293T cells were transfected with Myc-tagged NP and FLAG-tagged PB1 or PB2, together with Myc-CypE or Myc-CypE Δ137–186. Cell lysates were then immunoprecipitated with anti-FLAG antibody followed by immunoblotting with anti-Myc antibody. The data demonstrate that CypE impaired the interaction of NP with both PB1 and PB2 (**Figure 7**). These results further supported the deduction that CypE inhibits the replication and transcription of influenza A virus by impairing the formation of vRNP.

**Figure 8:**
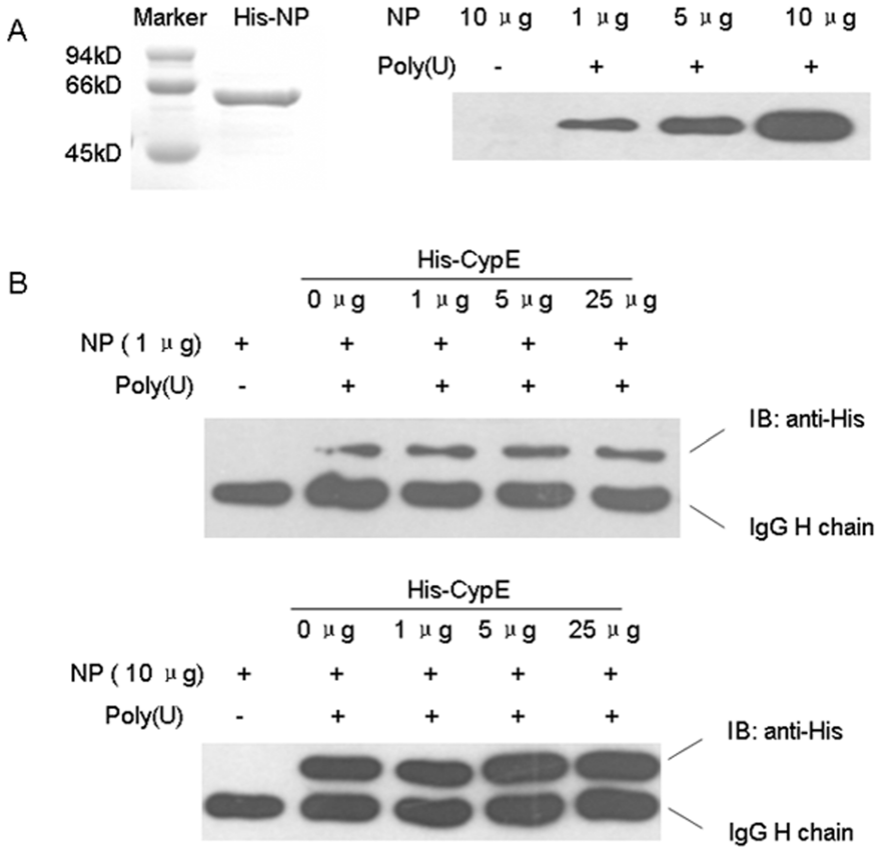
CypE has no effect on NP-RNA binding. (A) Purified NP and CypE proteins were ≥95% pure according to SDS-PAGE. Purified NP (1, 5, or 10 µg) was incubated with poly(U) agarose in 200 µl buffer, respectively, and blank agarose without poly(U) coupling was incubated with 10 µg NP as a negative control. (B) NP was incubated with different doses of CypE for 4 h at 4°C in 200 µl buffer. Then, poly(U)-agarose was incubated with the mixtures for 15 min at 4°C. His-NP bound to the agarose was detected by western blotting using an anti-His monoclonal antibody.

In addition, we examined whether CypE affects the binding of NP to RNA. His-tagged NP was incubated with poly(U)-agarose in the presence or absence of CypE, and then, the NP bound to the poly(U)-agarose was detected by immunoblotting with anti-His antibody. As shown in **Figure 8**, excessive CypE had no effect on the interaction of NP with RNA.

## Discussion

As is well known, hemagglutinin (HA) plays a critical role in receptor binding and membrane fusion during the entry stage of influenza A virus infection, and thus, the virus is able to access cells that display matched receptors on their surface to initiate infection. Though both SAα2, 6-Gal and SAα2, 3-Gal receptors are present on cells in many organs of birds, pigs, and humans [31], [32], [33], [34], the susceptibility of different host species to influenza A virus varies greatly, suggesting the existence of inhibitory mechanisms beyond the sialic acid receptor specificity. It has been hypothesized that as-yet undetermined host restriction factors could be involved in mediating cellular resistance to viral replication, reducing the efficiency of inter-species transmission. Thus, the identification of host factors with anti-influenza effects on viral replication may better provide insight into viral host adaptation. To date, several host factors have been identified that interact with the viral proteins/complexes and inhibit the replication of influenza A virus, implicating them as host barriers to limit viral replication [16], [24], [35], [36], [37], [38], [39]. Here, CypE was also proven to be an influenza A virus inhibitory factor.

It has been reported that the nuclear import of vRNPs is mediated through the interaction between NP and importin-α [15]. Moreover, the interaction between importin-α and PB2 or NP is a determinant of virus host range, and different importin-α isoforms play critical and distinct roles in host adaptation [14], [18], [40]. On the contrary, the current study demonstrated that the over-expression of CypE delays the translocation of NP from the nucleus to the cytoplasm (Figure 4E), suggesting that CypE may play an important role in mediating vRNP export through its interaction with NP. Our latest findings demonstrated that human CypE displays different binding affinities to NPs from several subtypes of influenza A viruses (data not shown). This finding further supports the conclusion that CypE is a restriction factor to viral infection and also demonstrates that CypE may play a role in viral host adaptation. Likewise, it will be important to investigate whether the tissue distribution of CypE is associated with the tissue tropism of influenza A virus. These findings will help us to better understand the molecular mechanisms of host restriction barriers, host adaptation and tissue tropism of influenza A virus.

It has been reported that CypA inhibits the replication of influenza A virus in the absence of its PPIase activity [24]. Here, we demonstrated that the antiviral function of CypE is also independent of its PPIase activity because the CypE R191A/W257A mutant, which is defective in PPIase activity [41], also equally inhibited the replication of influenza A virus (Figure S3). Furthermore, our mutagenesis data indicate that the key sites of PPIase activity (R191A and W257A) are not included in the CypE 137–186 domain responsible for its binding to NP. These data suggest that CypE may play roles in cells infected with influenza A virus that are independent of its PPIase activity.

NP forms oligomers as a purified recombinant protein, in virions and in infected cells [5], [30]. Furthermore, when the critical oligomerization sites (E339A, R416A, and Δ402–428) are substituted or deleted, all three mutants only form monomers [30]. Moreover, the E339A and R416A NP mutants lost the ability to support the vRNP activity, and these mutants result in growth defects in mutant viruses [29]. These previous reports indicate that NP oligomerization is a fundamental and necessary step for viral replication and transcription. In the present study, immunoprecipitation assays were employed to demonstrate that the over-expression of CypE interferes with NP self-association (Figure 6B), suggesting that the over-expression of CypE inhibits the initiation of viral replication and transcription. A similar method was also used to examine the effect of A20 on the dimerization of IRF-3 [42]. According to the binding domains of NP to CypE (**Figure S4**), the critical E339 residue for NP oligomerization is included in the domain responsible for CypE binding.

Elton *et al*. (1999) demonstrate that the NP R416A mutant, which only forms monomers [30], exhibits little or no observable RNA binding activity [43], suggesting that proper self-association is important for binding to RNA. However, the present study indicates that NP is bound to CypE and, therefore, not polymerized but retains RNA binding activity. It seems that there is a conflict between our report and Elton *et al*. (1999), but our results are similar to another report about NP-RNA binding [44]. Indeed, it has been shown that the NP Δ256–339 and Δ378–460 mutants, which lack critical oligomerization sites [30], display RNA binding activity [44]. There are two possible reasons for this discrepancy. One is that the systems used in the two reports are different. In Albo *et al*. (1995), polynucleotide was used to determine the RNA binding region in NP [44], but in Elton *et al*. (1999), the viral RNA was employed to identify the essential amino acid residues of NP required for RNA binding [43]. The other reason is that the R416 residue is also a relevant binding site to RNA. In the present study, we also used poly(U) to detect NP-RNA binding, and NP R416 is not included in the domain responsible for its interaction with CypE.

In conclusion, the findings described in the present study demonstrate that CypE exhibits an anti-influenza activity by targeting the functions of NP. The mechanism of its anti-influenza action occurs by impairing the formation of vRNP. Thus, CypE may play some role in host adaptation and tissue tropism for the infection of influenza A viruses. This study provides insight into genetic breeding for resistance to influenza A virus infection and discovery of novel anti-influenza drug targets.

## Materials and Methods

### Virus and Cells

Influenza A virus A/WSN/33 (H1N1) was propagated in the allantoic cavities of 10-day-old specific pathogen-free (SPF) embryonic chicken eggs at 37°C for 2 days. Then, the virions in the allantoic fluid were inoculated into the Madin-Darby Canine Kidney (MDCK) cell line (ATCC CCL-34). For plaque assay, MDCK cells were seeded in six-well tissue culture plates. The MDCK cells were infected with the influenza virus for different treatments for 1 h at 37°C. The virus inoculum was removed by washing with PBS. Cell monolayers were then overlaid with overlay medium (DMEM supplemented with 2% low-melting-point agarose and 2 µg/ml TPCK-treated trypsin) and incubated at 37°C. Visible plaques were counted at 4 days p.i., and the virus titers were determined. All data are expressed as the means of three independent experiments. MDCK, human embryonic kidney (293T) [45], HeLa (ATCC CCL-2), and A 549 (ATCC CCL-185) cell lines were cultured in DMEM medium supplemented with 10% fetal bovine serum.

### Antibodies

Polyclonal antibodies against NP and CypE were generated by immunizing rabbits and mice with purified His-tagged CypE and NP, respectively; the generation of antibodies was boosted three times by immunization with the proteins at 2-week interval. Anti-His tag monoclonal antibody, anti-β-actin polyclonal antibody, and anti-Myc tag monoclonal antibody were purchased from Santa Cruz Biotechnology. Anti-FLAG (M2) monoclonal antibody and anti-Myc polyclonal antibody were purchased from Sigma.

### Plasmid construction

The full-length NP gene obtained from A/WSN/33 virus was cloned into the pET30a (+) vector. CypE and its truncations (CypE 1–84, CypE 85–136, CypE 137–275, CypE 137–301, CypE 137–186, CypE 187–236, CypE 237–275, and CypE Δ137–186) were cloned into the pGEX-6p-1 vector. For co-immunoprecipitation, confocal, and luciferase assays, the NP gene was cloned into the pcDNA3-FLAG and pCMV-Myc vectors, respectively; the full-length CypE gene and CypE Δ137–186 were cloned into pCMV-Myc. The expression plasmids for the PA, PB1, and PB2 genes from A/WSN/33 virus were generated by cloning into the pCDNA3-FLAG vector.

### GST pull-down assays

GST-fused CypE and its truncations fused were purified using Sepharose 4B-glutathione (GE Healthcare). His-tagged NP and 200 µl lysate from MDCK cells infected with A/WSN/33 virus were incubated with equal amounts of GST alone and GST-CypE bound to glutathione-Sepharose 4B beads in binding buffer (1% NP-40, 150 mM NaCl, 20 mM HEPES (pH 7.4), 10% glycerol, and 1 mM EDTA) with protease inhibitor cocktail (Roche). The beads were washed five times with washing buffer (1% NP-40, 300 mM NaCl, 20 mM HEPES (pH 7.4), 10% glycerol, and 1 mM EDTA) with protease inhibitor cocktail. After extensive washing, the NP bound to the beads was extracted and analyzed by western blotting with an anti-His monoclonal antibody or an anti-NP polyclonal antibody.

### Co-immunoprecipitation assays

The 293T cells were transfected with different plasmids using lipofectamine 2000 (Invitrogen). At 48 h post-transfection (p.t.), the cells were lysed in binding buffer supplemented with protease inhibitor cocktail. The cell lysates were immunoblotted with anti-Myc or anti-FLAG antibody to confirm the expression of the genes of interest in the 293T cells, and then, the co-immunoprecipitation assays were performed using anti-FLAG M2 affinity gel. The immunoprecipitated proteins were assayed with anti-Myc polyclonal antibody. For the binding assay between endogenous CypE and NP during influenza virus infection, 293T cells were infected with A/WSN/33 (MOI = 1). At 12 h p.i., the cell lysates were subjected to co-immunoprecipitation assays using rabbit anti-NP antibody.

### Immunofluorescence assays

HeLa cells were washed three times with PBS, fixed in 4% paraformaldehyde for 30 min at room temperature, permeabilized with 0.5% Triton X-100 in PBS (PBST) for 20 min, and then incubated for 1 h with anti-CypE rabbit antiserum and anti-NP mouse antiserum. After washing with PBST, the cells were incubated for 1 h with the secondary antibodies. The cells were then observed under a Leica confocal microscope.

For influenza virus infectivity assays, 293T cells were transfected with 1 µg/well pCMV-Myc-CypE plasmid or 1 µg/well pCMV-Myc empty vector (as a control) and pEGFP-N1 (0.1 µg/well). At 12 h p.t., the cells were infected with influenza virus A/WSN/33 at an MOI of 1. At 6 and 8 h p.i., the cells were treated as described above and then stained with anti-Myc and anti-NP antibodies. The cells were observed under a Leica confocal microscope.

### RNA interference assays

The affective siRNA duplexes for CypE were si-CypE: 5′-ATTGTGGTTTGTGAAATCACCGCCC-3′ (Invitrogen). The 293T or A549 cells were transfected with si-CypE or si-Control according to manufacturer's instructions using RNAiMax transfection reagent (Invitrogen). At 48 h p.t., the cells were infected with influenza A virus A/WSN/33. The efficiency of CypE knock-down was confirmed by western blotting with the indicated antibodies. The virus titers in the media were measured by plaque assay.

### Quantitative real-time PCR assays

Transfected A549 cells were harvested, and total RNA was extracted for quantitative real-time PCR assays. We used published primers (vRNA (5′-AGCGAAAGCAGG-3′ and 5′-AGCAAAAGCAGG-3′), cRNA (5′-AGTAGAAACAAGG -3′), and mRNA (oligo (dT))) to detect the vRNA, cRNA, and mRNA for reverse transcription reactions [46]. The real-time PCR primers were as follows: WSN M1 (forward, 5′-TCTGATCCTCTCGTCATTGCAGCAA-3′; reverse, 5′-AATGACCATCGTCAACATCCACAGC-3′) [35]; and WSN NP (forward, 5′-TGGCACTCCAATTTGAATGATG-3′; reverse, 5′-TCCATTCCTGTGCGAACAAG-3′) [47]. GAPDH mRNA served as an internal control: (forward, 5′-GGTGGTCTCCTCTGACTTCAACA-3′; and reverse, 5′-GTTGCTGTAGCCAAATTCGTTGT-3′), as described in [48]. The PCR program was 95°C for 30 s followed by 40 cycles of 94°C for 5 s and 60°C for 30 s, and dissociation curve analysis of amplification products was performed at the end of each PCR reaction to confirm that only one PCR product was amplified and detected. Each sample was run in triplicate along with the internal control gene. Data analysis of real time PCR was performed with Rotor Gene 6000 Series Software (Corbett).

### Luciferase reporter assays for influenza polymerase complex activity

All polymerase complex component plasmids were co-transfected with a luciferase reporter plasmid that contained non-coding sequence from the NS segment of the influenza A virus genome and the luciferase gene that was driven by the PolI promoter into 293T cells. At the same time, pCMV-Myc empty vector (0.45 µg), different doses of CypE plasmid (0.05, 0.15, and 0.45 µg), and pCMV-CypE Δ137–186 (0.45 µg) were also transfected into 293T cells, respectively. At 36 h p.t., cell lysates were prepared using a luciferase assay kit (Promega), and the relative activities with different doses of CypE were compared. Plasmid pCMV-β-galactosidase, which expresses β-galactosidase, was co-transfected as an internal control for data normalization.

### NP-RNA binding assays

His-tagged NP and CypE were purified using Ni-NTA affinity agarose. NP was incubated with different doses of CypE (1, 5, and 25 µg) for 4 h at 4°C in a Tris-HCl buffer (pH 7.4) containing 1 U/µl RNase inhibitor. Then, equimolar amount of poly(U) agarose was incubated with the mixtures for 15 min at 4°C. At the same time, an equivalent amount of anti-FLAG M2 agarose was added to every binding reaction as an internal control. After washing extensively, the His-NP bound to the agarose was detected by western blotting using an anti-His monoclonal antibody.

## Supporting Information

Figure S1 **A549 cells were transfected with si-CypE or si-control and then infected with influenza virus A/WSN/33 at an MOI of 0.1.** The cell lysates were analyzed by western blotting with the indicated antibodies (A), and the viral titers of the media were measured by plaque assay (B). **, *p<0.01*.
(TIF)

Figure S2 **293T cells were transfected with 1 µg CypE and 1 µg pCMV-Myc vector as a control, and then they were infected with A/WSN/33 (MOI = 1).** The cRNA, vRNA, and mRNA levels of the NP and M1 genes were analyzed by quantitative real-time PCR after 2, 4, and 8 h p.i.. Error bars represented the SEM.
(TIF)

Figure S3 **293T cells were transfected with FLAG-CypE or FLAG-CypE R191A/W257A plasmid and then infected with influenza virus A/WSN/33 at an MOI of 0.1.** The cell lysates was analyzed by western blotting with the corresponding antibodies (A). The media were collected, and the viral titers were measured (B). Error bars represented the SD. *, p<0.05.
(TIF)

Figure S4 **FLAG-tagged NP plus CypE or its truncations were transfected into 293T cells.** The co-immunoprecipitation assays were performed using anti-FLAG M2 affinity gel. The immunoprecipitated proteins were assayed with an anti-Myc polyclonal antibody. “Input” shows ∼1/20 of the total protein.
(TIF)

## References

[1] Palese P (1977). The genes of influenza virus.. Cell.

[2] Murti KG, Webster RG, Jones IM (1988). Localization of RNA polymerases on influenza viral ribonucleoproteins by immunogold labeling.. Virology.

[3] Kingsbury DW, Jones IM, Murti KG (1987). Assembly of influenza ribonucleoprotein in vitro using recombinant nucleoprotein.. Virology.

[4] Pons MW, Schulze IT, Hirst GK, Hauser R (1969). Isolation and characterization of the ribonucleoprotein of influenza virus.. Virology.

[5] Ruigrok RW, Baudin F (1995). Structure of influenza virus ribonucleoprotein particles. II. Purified RNA-free influenza virus ribonucleoprotein forms structures that are indistinguishable from the intact influenza virus ribonucleoprotein particles.. J Gen Virol.

[6] Portela A, Digard P (2002). The influenza virus nucleoprotein: a multifunctional RNA-binding protein pivotal to virus replication.. J Gen Virol.

[7] Medcalf L, Poole E, Elton D, Digard P (1999). Temperature-sensitive lesions in two influenza A viruses defective for replicative transcription disrupt RNA binding by the nucleoprotein.. J Virol.

[8] Biswas SK, Boutz PL, Nayak DP (1998). Influenza virus nucleoprotein interacts with influenza virus polymerase proteins.. J Virol.

[9] Kingsbury DW, Webster RG (1969). Some Properties of Influenza Virus Nucleocapsids.. J Virol.

[10] Coloma R, Valpuesta JM, Arranz R, Carrascosa JL, Ortin J (2009). The structure of a biologically active influenza virus ribonucleoprotein complex.. PLoS Pathog.

[11] Duesberg PH (1969). Distinct subunits of the ribonucleoprotein of influenza virus.. J Mol Biol.

[12] Yamanaka K, Ishihama A, Nagata K (1990). Reconstitution of influenza virus RNA-nucleoprotein complexes structurally resembling native viral ribonucleoprotein cores.. J Biol Chem.

[13] Digard P, Elton D, Bishop K, Medcalf E, Weeds A (1999). Modulation of nuclear localization of the influenza virus nucleoprotein through interaction with actin filaments.. J Virol.

[14] Gabriel G, Herwig A, Klenk HD (2008). Interaction of polymerase subunit PB2 and NP with importin alpha1 is a determinant of host range of influenza A virus.. PLoS Pathog.

[15] O'Neill RE, Jaskunas R, Blobel G, Palese P, Moroianu J (1995). Nuclear import of influenza virus RNA can be mediated by viral nucleoprotein and transport factors required for protein import.. J Biol Chem.

[16] Wang P, Song W, Mok BW, Zhao P, Qin K (2009). Nuclear factor 90 negatively regulates influenza virus replication by interacting with viral nucleoprotein.. J Virol.

[17] Boivin S, Hart DJ (2011). Interaction of the influenza A virus polymerase PB2 C-terminal region with importin {alpha} isoforms provides insights into host adaptation and polymerase assembly.. J Biol Chem.

[18] Gabriel G, Klingel K, Otte A, Thiele S, Hudjetz B (2011). Differential use of importin-alpha isoforms governs cell tropism and host adaptation of influenza virus.. Nat Commun.

[19] Elton D, Simpson-Holley M, Archer K, Medcalf L, Hallam R (2001). Interaction of the influenza virus nucleoprotein with the cellular CRM1-mediated nuclear export pathway.. J Virol.

[20] Liao TL, Wu CY, Su WC, Jeng KS, Lai MM (2010). Ubiquitination and deubiquitination of NP protein regulates influenza A virus RNA replication.. EMBO J.

[21] Franke EK, Yuan HE, Luban J (1994). Specific incorporation of cyclophilin A into HIV-1 virions.. Nature.

[22] Luban J, Bossolt KL, Franke EK, Kalpana GV, Goff SP (1993). Human immunodeficiency virus type 1 Gag protein binds to cyclophilins A and B.. Cell.

[23] Yang F, Robotham JM, Nelson HB, Irsigler A, Kenworthy R (2008). Cyclophilin A is an essential cofactor for hepatitis C virus infection and the principal mediator of cyclosporine resistance in vitro.. J Virol.

[24] Liu X, Sun L, Yu M, Wang Z, Xu C (2009). Cyclophilin A interacts with influenza A virus M1 protein and impairs the early stage of the viral replication.. Cell Microbiol.

[25] Xu C, Meng S, Liu X, Sun L, Liu W (2011). Chicken cyclophilin A is an inhibitory factor to influenza virus replication.. Virol J.

[26] Watashi K, Ishii N, Hijikata M, Inoue D, Murata T (2005). Cyclophilin B is a functional regulator of hepatitis C virus RNA polymerase.. Mol Cell.

[27] Mi H, Kops O, Zimmermann E, Jaschke A, Tropschug M (1996). A nuclear RNA-binding cyclophilin in human T cells.. FEBS Lett.

[28] Chan WH, Ng AK, Robb NC, Lam MK, Chan PK (2010). Functional analysis of the influenza virus H5N1 nucleoprotein tail loop reveals amino acids that are crucial for oligomerization and ribonucleoprotein activities.. J Virol.

[29] Li Z, Watanabe T, Hatta M, Watanabe S, Nanbo A (2009). Mutational analysis of conserved amino acids in the influenza A virus nucleoprotein.. J Virol.

[30] Ye Q, Krug RM, Tao YJ (2006). The mechanism by which influenza A virus nucleoprotein forms oligomers and binds RNA.. Nature.

[31] Kuchipudi SV, Nelli R, White GA, Bain M, Chang KC (2009). Differences in influenza virus receptors in chickens and ducks: Implications for interspecies transmission.. J Mol Genet Med.

[32] Kogure T, Suzuki T, Takahashi T, Miyamoto D, Hidari KI (2006). Human trachea primary epithelial cells express both sialyl(alpha2–3)Gal receptor for human parainfluenza virus type 1 and avian influenza viruses, and sialyl(alpha2–6)Gal receptor for human influenza viruses.. Glycoconj J.

[33] Shinya K, Ebina M, Yamada S, Ono M, Kasai N (2006). Avian flu: influenza virus receptors in the human airway.. Nature.

[34] Nelli RK, Kuchipudi SV, White GA, Perez BB, Dunham SP (2010). Comparative distribution of human and avian type sialic acid influenza receptors in the pig.. BMC Vet Res.

[35] Watanabe K, Fuse T, Asano I, Tsukahara F, Maru Y (2006). Identification of Hsc70 as an influenza virus matrix protein (M1) binding factor involved in the virus life cycle.. FEBS Lett.

[36] Li S, Min JY, Krug RM, Sen GC (2006). Binding of the influenza A virus NS1 protein to PKR mediates the inhibition of its activation by either PACT or double-stranded RNA.. Virology.

[37] Min JY, Krug RM (2006). The primary function of RNA binding by the influenza A virus NS1 protein in infected cells: Inhibiting the 2′–5′ oligo (A) synthetase/RNase L pathway.. Proc Natl Acad Sci U S A.

[38] Nemeroff ME, Barabino SM, Li Y, Keller W, Krug RM (1998). Influenza virus NS1 protein interacts with the cellular 30 kDa subunit of CPSF and inhibits 3′end formation of cellular pre-mRNAs.. Mol Cell.

[39] Opitz B, Rejaibi A, Dauber B, Eckhard J, Vinzing M (2007). IFNbeta induction by influenza A virus is mediated by RIG-I which is regulated by the viral NS1 protein.. Cell Microbiol.

[40] Boivin S, Hart DJ (2011). Interaction of the influenza A virus polymerase PB2 C-terminal region with importin {alpha} isoforms provides insights into host adaptation and polymerase assembly.. J Biol Chem.

[41] Wang T, Yun CH, Gu SY, Chang WR, Liang DC (2005). 1.88 A crystal structure of the C domain of hCyP33: a novel domain of peptidyl-prolyl cis-trans isomerase.. Biochem Biophys Res Commun.

[42] Saitoh T, Yamamoto M, Miyagishi M, Taira K, Nakanishi M (2005). A20 is a negative regulator of IFN regulatory factor 3 signaling.. J Immunol.

[43] Elton D, Medcalf L, Bishop K, Harrison D, Digard P (1999). Identification of amino acid residues of influenza virus nucleoprotein essential for RNA binding.. J Virol.

[44] Albo C, Valencia A, Portela A (1995). Identification of an RNA binding region within the N-terminal third of the influenza A virus nucleoprotein.. J Virol.

[45] DuBridge RB, Tang P, Hsia HC, Leong PM, Miller JH (1987). Analysis of mutation in human cells by using an Epstein-Barr virus shuttle system.. Mol Cell Biol.

[46] Liang Y, Huang T, Ly H, Parslow TG, Liang Y (2008). Mutational analyses of packaging signals in influenza virus PA, PB1, and PB2 genomic RNA segments.. J Virol.

[47] Goodman AG, Smith JA, Balachandran S, Perwitasari O, Proll SC (2007). The cellular protein P58IPK regulates influenza virus mRNA translation and replication through a PKR-mediated mechanism.. J Virol.

[48] Lai JP, Yang JH, Douglas SD, Wang X, Riedel E (2003). Quantification of CCR5 mRNA in human lymphocytes and macrophages by real-time reverse transcriptase PCR assay.. Clin Diagn Lab Immunol.

