# Supplementary figures and images for: Cyclophilin E Functions as a Negative Regulator to Influenza Virus Replication by Impairing the Formation of the Viral Ribonucleoprotein Complex
Source: PLoS One. 2011 Aug 24;6(8):e22625. doi: 10.1371/journal.pone.0022625 (PMC3160840; doi:10.1371/journal.pone.0022625)

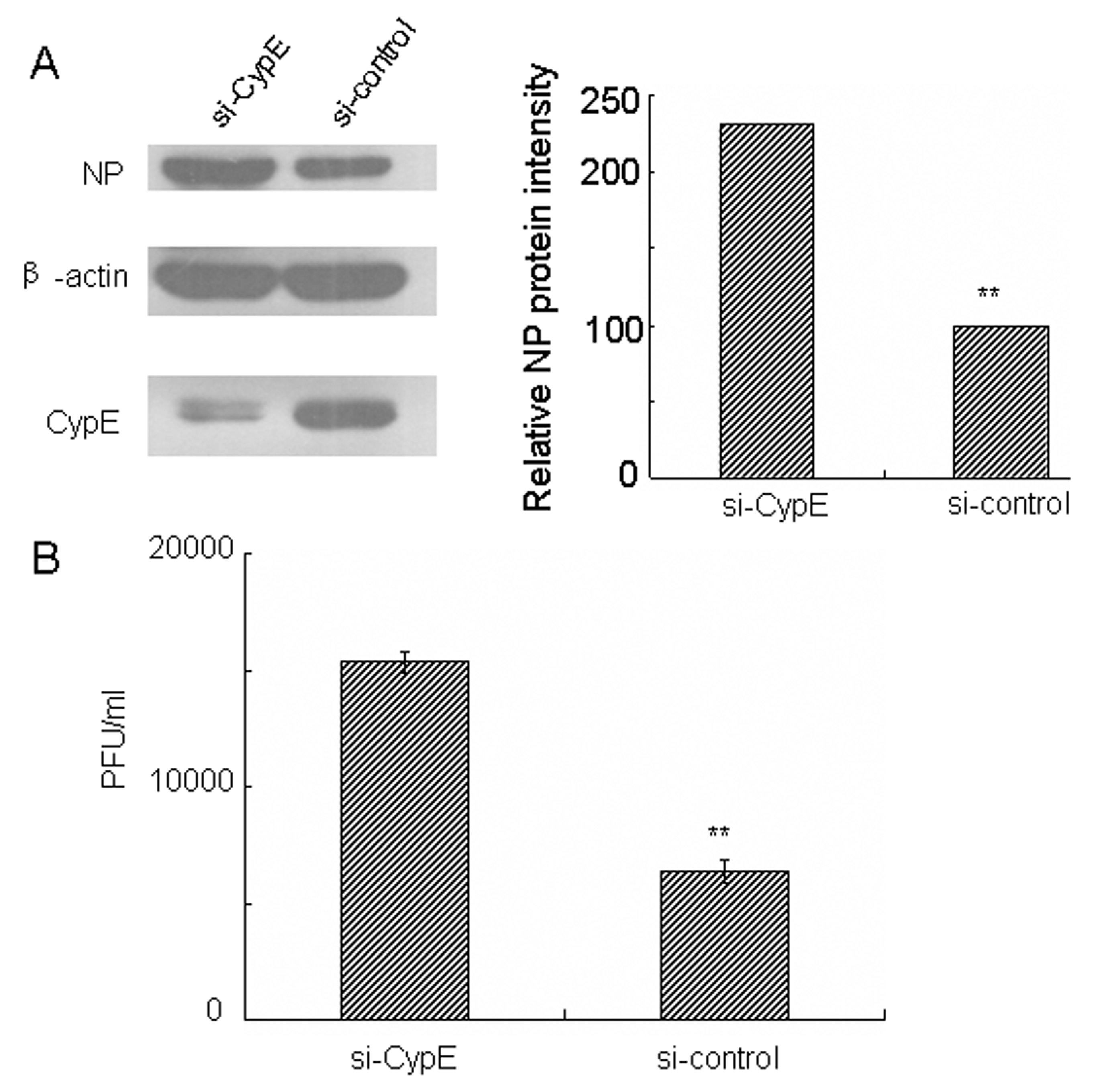

Supplement: Figure S1 — A549 cells were transfected with si-CypE or si-control and then infected with influenza virus A/WSN/33 at an MOI of 0.1. The cell lysates were analyzed by western blotting with the indicated antibodies (A), and the viral titers of the media were measured by plaque assay (B). **, p<0.01. (TIF) [file pone.0022625.s001.tif]

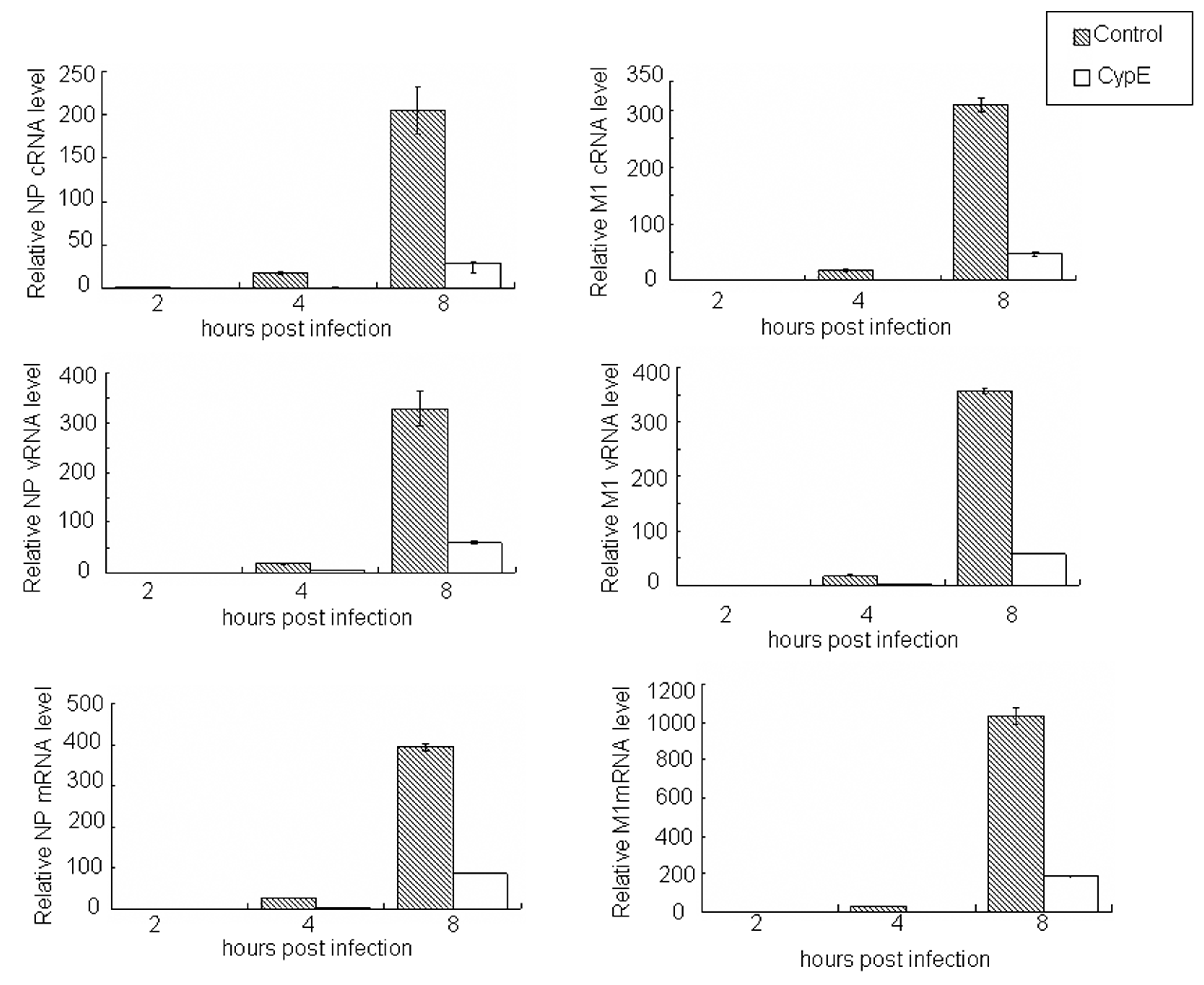

Supplement: Figure S2 — 293T cells were transfected with 1 µg CypE and 1 µg pCMV-Myc vector as a control, and then they were infected with A/WSN/33 (MOI = 1). The cRNA, vRNA, and mRNA levels of the NP and M1 genes were analyzed by quantitative real-time PCR after 2, 4, and 8 h p.i.. Error bars represented the SEM. (TIF) [file pone.0022625.s002.tif]

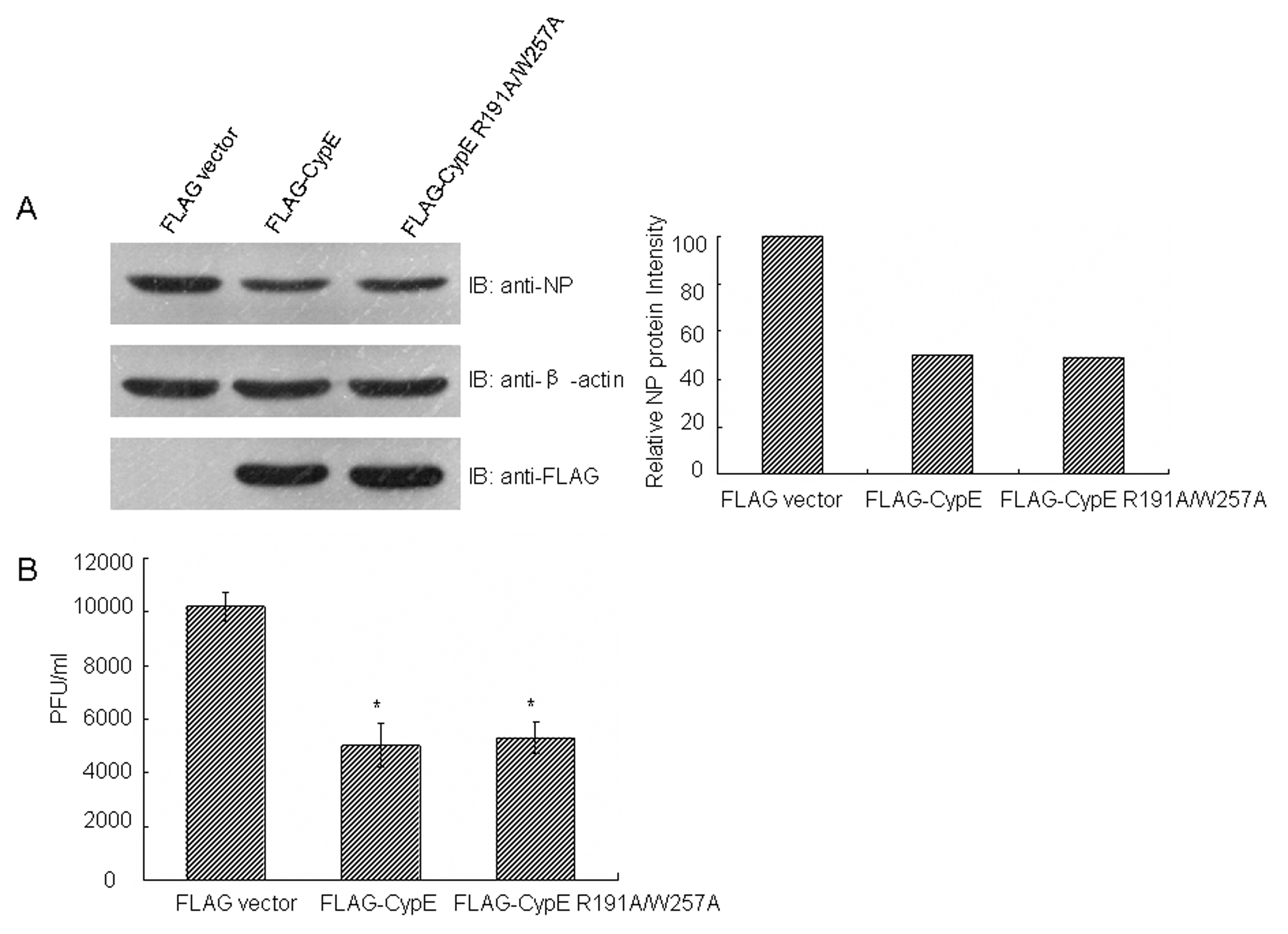

Supplement: Figure S3 — 293T cells were transfected with FLAG-CypE or FLAG-CypE R191A/W257A plasmid and then infected with influenza virus A/WSN/33 at an MOI of 0.1. The cell lysates was analyzed by western blotting with the corresponding antibodies (A). The media were collected, and the viral titers were measured (B). Error bars represented the SD. *, p<0.05. (TIF) [file pone.0022625.s003.tif]

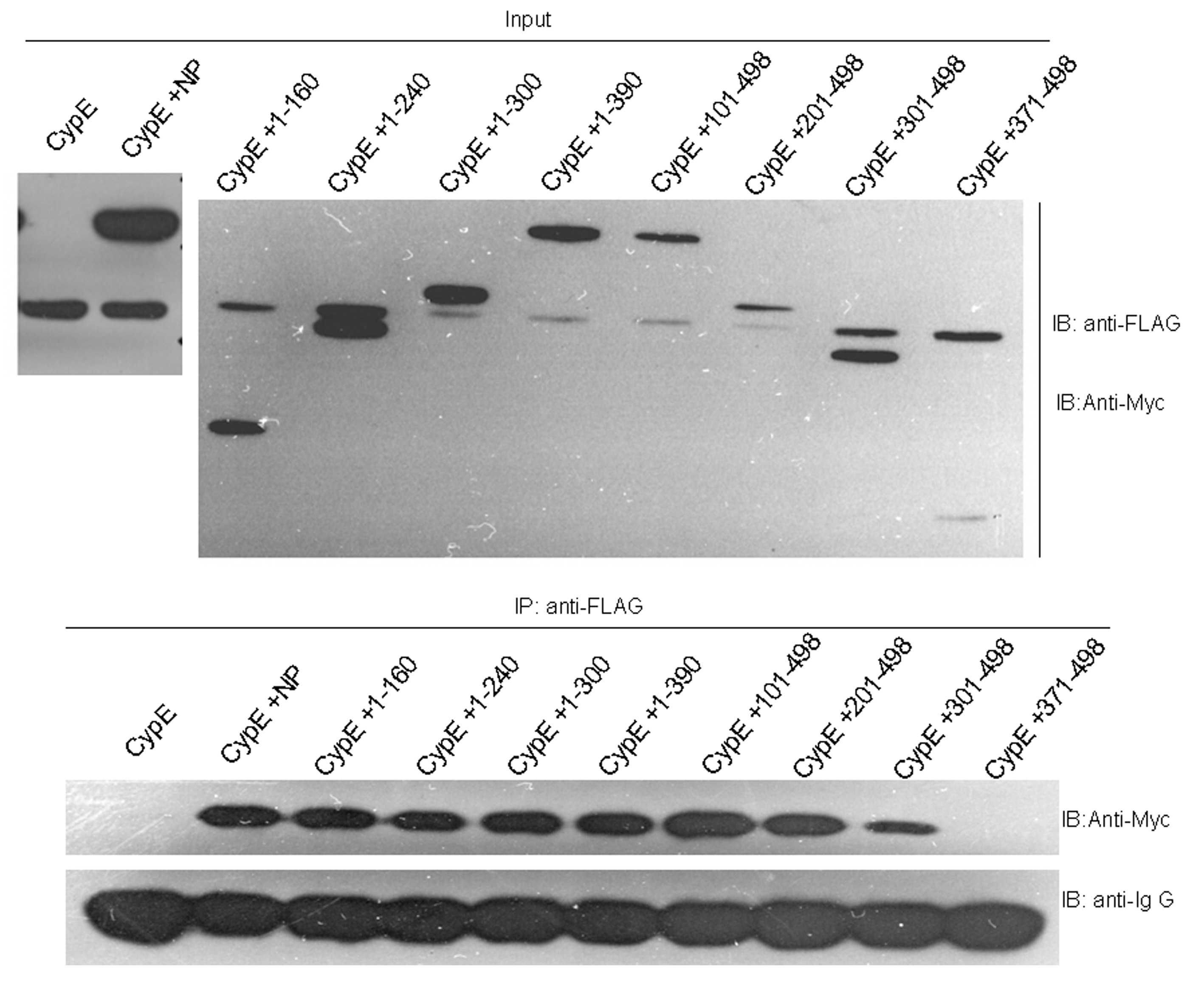

Supplement: Figure S4 — FLAG-tagged NP plus CypE or its truncations were transfected into 293T cells. The co-immunoprecipitation assays were performed using anti-FLAG M2 affinity gel. The immunoprecipitated proteins were assayed with an anti-Myc polyclonal antibody. “Input” shows ∼1/20 of the total protein. (TIF) [file pone.0022625.s004.tif]
